# The complete mitochondrial genome of *Dendrophyllia minuscula* (Cnidaria: Scleractinia) from the NEOM region of the Northern Red Sea

**DOI:** 10.1080/23802359.2022.2074803

**Published:** 2022-05-12

**Authors:** Tullia I. Terraneo, Roberto Arrigoni, Fabio Marchese, Giovanni Chimienti, Ameer Abdulla Eweida, Mattie Rodrigue, Francesca Benzoni

**Affiliations:** a Red Sea Research Center, King Abdullah University of Science and Technology, (KAUST), Thuwal, Kingdom of Saudi Arabia; b Department of Biology and Evolution of Marine Organisms (BEOM), Genoa Marine Centre (GMC), Stazione Zoologica Anton Dohrn–National Institute of Marine Biology, Ecology and Biotechnology, Villa del Principe, Genoa, Italy; c Department of Biology, University of Bari Aldo Moro, Bari, Italy; d CoNISMa, Rome, Italy; e Marine Conservation Division, Nature Conservation Authority, Neom, Saudi Arabia; f Rosenstiel School of Marine and Atmospheric Sciences, University of Miami, Miami, Florida, USA; g OceanX, New York, NY, USA

**Keywords:** Dendrophylliidae, mesophotic coral ecosystems, mitogenome, NGS, RAD sequencing

## Abstract

The scleractinian coral family Dendrophylliidae is a major component of shallow and deep-water coral ecosystems worldwide, but our knowledge on the evolutionary history of the family remains scarce. Here, we used ezRAD coupled with Illumina sequencing technology and reconstructed the complete mitochondrial genome of *Dendrophyllia minuscula* (GenBank accession number OL634845), from mesophotic depths in the Red Sea NEOM area. The mitochondrial genome of *D. minuscula* consisted of 19,054 bp, organized in 13 protein-coding genes, 2 rRNA genes, and 2 tRNA genes, in agreement with the Scleractinia typical mitogenome organization. This complete mitochondrial genome contributes toward a better knowledge of mesophotic and deep-water coral diversity and evolutionary history.

The family Dendrophylliidae Gray, 1847 is one of the most diverse families of scleractinian corals worldwide (Cairns 2001), comprising 25 extant accepted genera and 185 species (Hoeksema and Cairns 2021). It is a major component of shallow reef and mesophotic ecosystems as well as deep-water coral bioherms (Cairns 2001, 2007; Loya et al. 2019). Although the ecological role of Dendrophylliidae is well known, our current knowledge about the phylogenetic relationships of the family remains scarce. Most taxa in fact have been predominately investigated morphologically or with few genes (Arrigoni et al. 2014; Kitahara et al. 2016; Capel et al. 2020). Reduced-representation genome approaches, such as RAD sequencing, are shedding light into coral evolution by providing a fast and efficient way to reconstruct mitochondrial genomes (hereafter mitogenome) (Forsman et al. 2017; Terraneo et al. 2018a, 2018b).

Providing habitat for several benthic animals, such as bryozoans, serpulids, foraminifera, and smaller ahermatypic corals, *Dendrophyllia minuscula* Bourne, 1905 is a main framework builder in the Red Sea lower mesophotic and deep waters (Fricke and Hottinger 1983; Fricke and Schuhmacher 1983; Taviani et al. 2007). This basin harbors a diverse benthic fauna adapted to living in a highly oligotrophic environment, with water temperature stabilizing at 21 °C down to 2500 m depth, high salinity (>40), and low oxygen concentrations (<2 mg l^−1^) (Roder et al. 2013; Berumen et al. 2019).

During the Red Sea Deep Blue Expedition in 2020, *D. minuscula* was observed and collected at multiple sites in the NEOM region (*NEO*-from Latin ‘new’, *M*-from Arabic ‘future’), a new destination which is being developed in north-western Saudi Arabia. The specimen examined here lived in the Saudi Arabian Gulf of Aqaba (GPS coordinates: 29.264561 N, 34.927498 E) at 146.6 m depth. It was collected using an Argus Mariner XL ROV launched from the R/V OceanXplorer. IACUC standards and procedure were followed, which included ethical approval exemption for lower invertebrate species. Research and sampling permits were granted by NEOM and regulated by the Kingdom of Saudi Arabia. Frame grabs of the living colony were extracted from the ROV videos using MPC-HC (https://github.com/mpc-hc/mpc-hc). The specimen (voucher number CHR0019-6A) was identified by Professor Benzoni F and is archived at King Abdullah University of Science and Technology (KAUST, Saudi Arabia). We extracted genomic DNA using DNeasy^®^ Blood and Tissue Kit (Qiagen Inc., Hilden, Germany). Frequent cutting enzymes Mbol and Sau3AI (New England Biolabs, Ipswich, MA, USA) were used for DNA digestion following Toonen et al. (2013). ezRAD libraries were prepared using TruSeq^®^ Nano DNA kit (Illumina, San Diego, CA, USA) following the manufacture’s protocol. Paired-end sequencing was performed with NovaSeq6000 platform (Illumina, San Diego, CA, USA) at KAUST. Reads were assembled to *Dendrophyllia arbuscula* van der Horst, 1922 mitogenome (NC027590 - Japan) (Luz et al. 2016) using GeneiousPrime^®^ 2021.2.2 (Biomatters Ltd. Auckland, New Zealand), and a consensus sequence was exported using 0% majority option for coverage > 3 X. Genes were annotated using MITOS web-server (Bernt et al. 2013) and subsequentially manually verified.

The complete mitogenome of *D. minuscula* consisted of 19,054 bp, with the typical A + T rich base composition of Scleractinia mitogenomes (Fukami and Knowlton 2005; Medina et al. 2006). The following overall base composition was recovered: A 25.4%, T 37.3%, C 13.6%, and G 23.7%. The mitogenome of *D. minuscula* presented 97.4% pairwise similarity to the mitogenome of *D. arbuscula* with the majority of SNPs retrieved in protein-coding genes. It included 13 protein-coding genes (PCGs), 2 ribosomal RNA genes (*rnl* and *rns*), and 2 transfer RNA genes (*trnM* and *trnW*), showing the typical gene order of scleractinian corals mitogenomes (Fukami and Knowlton 2005; Medina et al 2006). The PCG *nad6* started with ATA codon, *nad5*, *nad4L*, and *nad3* with GTG codon, whereas the remaining nine PCGs started with ATG codon. All PCGs showed either TAA or TAG as stop codons. The PCGs *nad5* and *cox1* were interrupted by group I introns. In particular, *nad5* intron included ten PCGs and *rns* for a total of 11,283 bp while *cox1* intron was 964 bp long, as reported for several scleractinian mitogenomes (Fukami et al. 2007).

Multiple alignment of the complete mitogenomes of *D. minuscula* and other representatives of Dendrophylliidae was performed with MAFFT 7.490 (Katoh and Standley 2013), resulting in a final length of 24,648 bp. The phylogenetic position of *D. minuscula* within the family was reconstructed using RAxML 2 (Stamatakis 2014) on the online CIPRES server (Miller et al. 2010), using the GRT + GAMMA model and 1000 rapid bootstrap replicates. The phylogeny reconstruction was rooted with *Porites* (Kitahara et al. 2016). The obtained phylogenetic tree showed that *D. minuscula* clustered within Dendrophylliidae and was sister to *D. arbuscula* (Figure 1). Future work should focus on the inclusion of mitogenomes from other Dendrophylliidae taxa not yet represented to better investigate the evolutionary history of the organisms ascribed to this ecologically important coral family.

**Figure 1.**
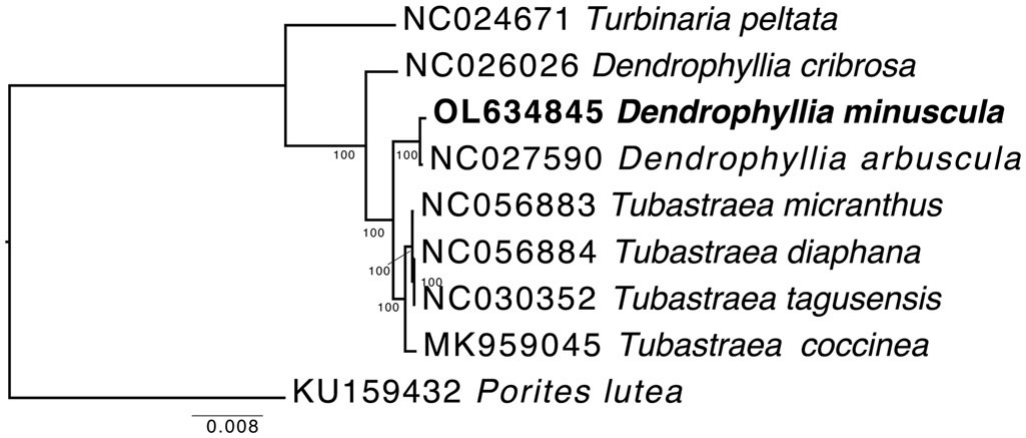
Phylogenetic reconstruction of *Dendrophyllia minuscula* and other representative taxa of the family Dendrophylliidae based on complete mitochondrial genomes. Numbers at nodes represent maximum likelihood bootstrap values. *Porites lutea* was selected as outgroup.

## Data Availability

The genome sequence data that support the findings of this study are openly available in GenBank of NCBI at (https://www.ncbi.nlm.nih.gov/nuccore/2175794262) under the accession no. OL634845. The associated **BioProject**, **SRA**, and **Bio-Sample** numbers are PRJNA779577, SRR17026845, and SAMN23423813, respectively.
